# RNAi Crop Protection Advances

**DOI:** 10.3390/ijms222212148

**Published:** 2021-11-10

**Authors:** Alejandro Hernández-Soto, Randall Chacón-Cerdas

**Affiliations:** 1 Doctorado en Ciencia Naturales para el Desarrollo (DOCINADE), Instituto Tecnológico de Costa Rica, Universidad Nacional, Universidad Estatal a Distancia, Cartago P.O. Box 159-7050, Costa Rica; 2 Costa Rica Institute of Technology, Biology School, Biotechnology Research Center, Cartago P.O. Box 159-7050, Costa Rica; rchacon@tec.ac.cr

**Keywords:** RNAi, dsRNA, silencing, encapsulation, liposomes, virus-like particles, polyplex nanoparticles, bioclay, regulatory

## Abstract

RNAi technology is a versatile, effective, safe, and eco-friendly alternative for crop protection. There is plenty of evidence of its use through host-induced gene silencing (HIGS) and emerging evidence that spray-induced gene silencing (SIGS) techniques can work as well to control viruses, bacteria, fungi, insects, and nematodes. For SIGS, its most significant challenge is achieving stability and avoiding premature degradation of RNAi in the environment or during its absorption by the target organism. One alternative is encapsulation in liposomes, virus-like particles, polyplex nanoparticles, and bioclay, which can be obtained through the recombinant production of RNAi in vectors, transgenesis, and micro/nanoencapsulation. The materials must be safe, biodegradable, and stable in multiple chemical environments, favoring the controlled release of RNAi. Most of the current research on encapsulated RNAi focuses primarily on oral delivery to control insects by silencing essential genes. The regulation of RNAi technology focuses on risk assessment using different approaches; however, this technology has positive economic, environmental, and human health implications for its use in agriculture. The emergence of alternatives combining RNAi gene silencing with the induction of resistance in crops by elicitation and metabolic control is expected, as well as multiple silencing and biotechnological optimization of its large-scale production.

## 1. Introduction

The world is moving toward a more sustainable crop production system that requires specific and efficient tools to battle plant pathogens. RNAi can be used for such purposes. The molecule is used in nature, degrades quickly, can disrupt the pathogen at a genetically specific level, and can complement the current agronomic crop protection practices used for organic, conventional, ecological, or technological production [1]. The reader may be familiar with the concept of DNA and genes located in the nucleus of eukaryote cells, containing the instructions to create organic molecules, mainly proteins. RNA messenger works as an intermediator, carrying the nucleus’s message to the cytoplasm to be read by the ribosomes to assemble the protein. The RNAi eukaryotic machinery is a complex system for virus defense and gene expression control, sometimes called post transcriptional gene silencing (PTGS). The system can be triggered by external specific dsRNA, resulting in its RNA messenger being blocked before it gets to the ribosome, leaving the organism, such as a pathogen, disarmed [2]. The extravesical delivery of dsRNA to disarm the expression system was proven to be natural and bidirectional from plant to fungal pathogens and vice versa cross-kingdom communication [3,4,5,6,7,8].

Consequently, RNAi represents an opportunity to emulate or improve the natural plant pathogen control system by providing well-designed external dsRNA [9]. Here, we aimed to present advantages in crop protection mediated by RNAi. There are two RNAi plant-based technologies: host-induced gene silencing (HIGS) since the 1990s and emerging spray-induced gene silencing (SIGS). Both can provide sustainable solutions to control pathogens, such as insects, viruses, and fungi. We will focus on SIGS because it is becoming an emerging affordable option with a cost reduction of approximately USD 0.5–1 per gram [10]; the small amount of dsRNA necessary seems to be near 2–10 g per hectare; it is safe; and it has fast environmental degradation [11,12,13]. When dsRNA is applied externally to plants, one proposed mechanism is that plant cells can take it and use it directly to tackle the pathogen through secreted vesicles containing the RNA at the site of the infection and plasmodesmata [14,15,16]. The amount of sprayed RNA may vary depending on the target species’ sensitivity to RNAi, the capacity to trigger the defense system, and the efficiency of the delivery method. Other challenges for this technology are the need for science-based risk assessment procedures for topical RNAi applications within existing plant protection product legislation, regulatory approaches [12,13], and strategies to use more than one target sequence to avoid resistance of uptake [17].

## 2. How it Works

The system was discovered in 1998 when the nematode *Caenorhabditis elegans* exposed to a double-stranded RNA injection resulted in gene silencing [18]. The finding received a Nobel Prize award in Physiology and Medicine in 2006. The cascade of reactions where the introduced dsRNA ends up knocking out other RNAs is fascinating and complex. The native system is microRNA and acts via PTGS, orchestrating internal gene expression when needed. It starts with genomic codifying sequences transcribed by RNApolII, producing the first double-stranded RNA pri-miRNA processed in the nucleus by several enzymes, such as DROSHA and DGCR8, into premiRNA. The molecule is exported to the cytoplasm by Exportin-5 and, with the help of several enzymes (helicase, Dicer, TRBP, and Agr-2 TNRC6), ends up in a piece of activated machinery “RISC complex” capable of repressing, destroying, or even inactivating genes at the genetic level based on the resulting RNA single-stranded template [1,19,20,21].

**Figure 1:**
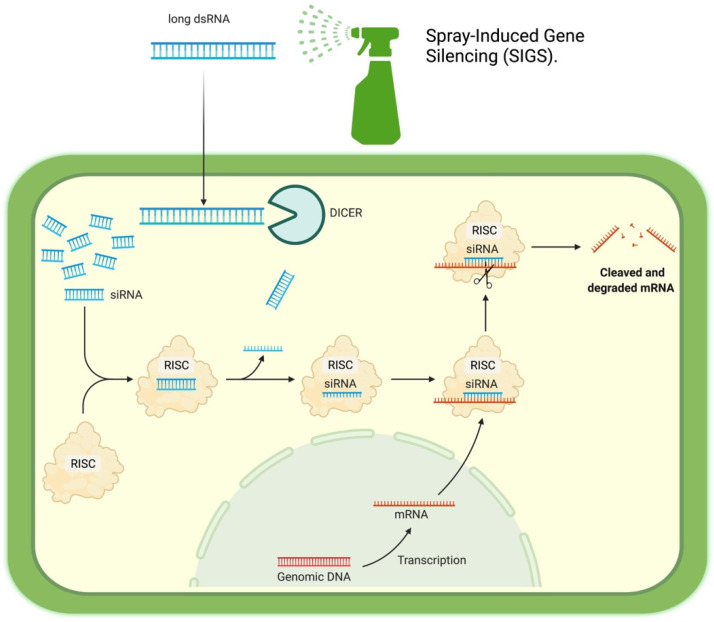
Spray-induced gene silencing (SIGS) mechanism.

What is extraordinary is how the RISC complex evolved to become a defense system capable of using external dsRNA sequences, known as the short interference RNA system (siRNA). The defense system usually works for viral defense. Scientists took advantage of this approach when adding external dsRNA to study gene expression in the model organism *C. elegans* [18,22]. Long dsRNA given to the nematode triggers the system. The first enzyme, Dicer, recognizes the molecule and cuts it into smaller fragments of 20–40 nts. The RISC complex can then separate the strands and use them to silence a complementary messenger RNA. Fungi and plants produce their dsRNA to disarm each other in a cross-kingdom communication fight. Even more fascinating is that plants can move external dsRNA from cell to cell and deliver vesicles with RNA targeted against fungi [7,14]. Figure 1 shows a diagram of the SIGS mechanism mentioned above.

## 3. Potential Targets

The potential targets of RNAi can be viruses, fungi, bacteria, nematodes, and endogenous genes. Next, we describe a general table (Table 1) containing several targets to demonstrate that the technology is flexible enough to start exploring other plagues, and broader reviews exist in case of interest by the reader [23]. Out of these possibilities, we would like to remark on the potential of using SIGS to produce a new generation of crop protection-specific products.

## 4. Encapsulation Technology to Improve Efficiency

The use of encapsulation technology has improved the effectiveness of gene silencing by designated RNAi. It confers protection and stability to dsRNA, preventing it from undergoing enzymatic or pH degradation while it is transported to the target cells where the release of dsRNA and its subsequent transformation into siRNA are required [44,45,46]. The development of the encapsulation system is related to the target organism, the type of RNAi to be delivered, and its uptake mechanism. According to this review, it is more common to find encapsulation of dsRNA for the control of insects by the oral route since they have an alkaline pH in the intestine and the presence of RNases in their digestive tract that would degrade naked RNAi [47]. A similar case occurs for nematodes, with the difference that the pH in their intestine is acidic [22]. Research in plants and fungi has shown that they are receptive to dsRNA and siRNA [3,28,48]. However, in insects, it has been proposed that silencing is more efficient when long dsRNA (>50 bp) is used compared to that when using sRNA, partly related to the selectivity of its incorporation mechanism [49].

Encapsulation can be produced by engineered microorganisms or synthetic micro/nanoparticles [50] using different materials, such as proteins [51], biopolymers [52], clays [44], or lipids [53]. Using these materials confers valuable properties for integrating siRNAs into target cells; for instance, capsid proteins that are already recognized by the target organism facilitate the penetration of dsRNAs into their cells, taking advantage of natural infection mechanisms [54]. There are multiple reports of engineered encapsulation systems that transport and protect RNAi, suitable crop protection applications (Table 2).

**Figure 2:**
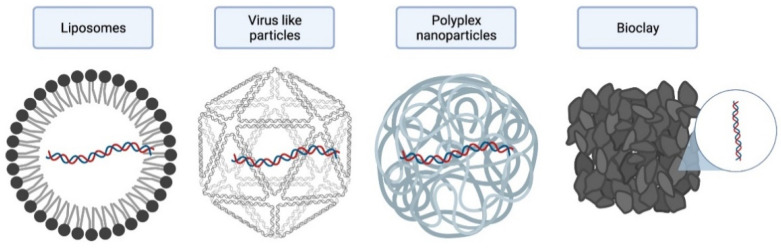
Primary encapsulation system for RNAi delivery.

According to the data available in the references, we identified four principal encapsulation systems: the formation of liposomes, virus-like particles, polyplex nanoparticles, and bioclay. As mentioned in Table 2, these systems coincide in the biodegradability of the materials, the ability to improve the stability of RNAi, and its synergistic effect to induce control. Figure 2 presents a diagram of the most reported systems.

### 4.1. Liposomes

Liposomes are spherical artificial vesicles synthesized from cholesterol and nontoxic natural phospholipids. They present hydrophilic and hydrophobic characteristics (amphiphilic molecules) that facilitate their interaction in multiple chemical environments [57]. The hydrophobic part consists of two fatty acid chains (10–24 carbon atoms and 0–6 double bonds in each chain), while the hydrophilic section is mainly phosphoric acid bound to a water-soluble molecule [58]. They are made up of at least one phospholipid layer. The type of lipid defines properties such as surface charge, solubility, and size of the vesicles; therefore, it is critical in the functionality of the particle. For example, unsaturated phosphatidylcholine lipids form much more permeable but less stable bilayers, while saturated phospholipids with long acyl chains form more rigid and waterproof structures. According to their size, they are classified as very small (0.025 μm) or large (2.5 μm) vesicles [57]. Multiple types of lipids are used as raw materials, the most frequent in the literature being phosphatidylglycerol (PG), phosphatidylcholine (PC), distearoylphosphatidylcholine (DSPC), dipalmitoylphosphatidylcholine (DPPC), dicetylphosphate, cholesterol (CH) stearylamine, or their mixture [59].

One advantage of this type of particle is its semipermeable character that simulates biological membranes, allowing smart delivery of the compounds it carries due to its cellular penetration capacity [60]. Additionally, its low solubility in hydrophilic environments is an advantage when looking for a slow-release mechanism, which helps immobilize compounds and facilitate their environmental biodegradation. However, this technology also has some technical disadvantages, such as a short half-life due to oxidation and hydrolysis of phospholipids, vulnerability to temperature, and others related to its cost of production [57]. The most cited synthesis methods are injection, electroformation, reverse-phase evaporation, hydration or thin-film hydration (Bangham method), microfluidics, freeze drying of double emulsions, heated membrane extrusion, detergent depletion, and supercritical fluid preparation. The challenge of the methods is to achieve stable vesicles with controlled and functional sizes [59,61].

The applications of liposomes are very versatile; for example, in the case of human and animal cells, they include a vast inventory of drug delivery systems to treat multiple diseases by reducing collateral damage in nontarget tissues and excessive doses [60]. In food science, they are used for the protection and controlled delivery of enzymes, vitamins, functional compounds, antibiotics, and metabolites to improve the properties of food [62]. In plants, in addition to the reports in Table 2 for RNAi delivery, they are used as carriers of CRISPR/Cas gene-editing systems, proteins, DNA, or mRNA [63]. Additionally, lipofection (liposome-mediated DNA delivery) in protoplasts [64] and nutrient transport/internalization are cited [65].

### 4.2. Virus Like-Particles (VLPs)

Virus-like particles (VLPs) are defined as supramolecular self-assemblies of proteins of approximately 10–200 nm in diameter, presenting the same or similar structure as that of native virions. VLPs are not infectious since they lack infective genetic material [66]. In addition to being nanocarriers, VLPs can be functionalized on the surface by ligand proteins, exhibiting additional properties such as immunological and labeling reactivity to changes in the chemical environment [67,68].

VLPs can be produced by heterologous expression systems such as baculovirus, bacteria, yeast, plants, and animal cells (insects, mammals, etc.). The expression vector to be used must be selected depending on the type of the desired protein and the target organism since the system’s success may depend on post-translational modifications. According to the type of virus from which they are derived, VLPs are classified as single-stranded RNA positive-sense viruses, single-stranded RNA negative-sense viruses, double-1 stranded RNA viruses, and single- and double-1 stranded DNA viruses. Additionally, VLPs can be produced in crude protein extracts (cell-free system), mainly when heterologous expression implies the production of toxic compounds for the vector [69]. Furthermore, the expression systems have different yields and are generally more efficient in viral and bacterial vectors. VLP surface conjugation can be covalent and noncovalent; the first option is to functionalize VLPs with large molecules, even complete proteins. This type of conjugation is frequent through bacteriophage expression systems (MS2 and Q-beta) [66]. Regarding noncovalent conjugation, a biotinylation process is required, which needs a linking agent such as streptavidin to ensure surface fixation [70].

Some of the advantages of VLPs are self-assembly, repetitive structural stability, and the resulting polydispersity systems. This improves uptake by target cells compared to that of naked molecules [71,72]. Using cell recognition proteins represents lower barriers to biointeraction [54]. However, its production is not exempt from limitations related to vectors and strategic aspects in terms of system complexity, speed of expression, performance, scalability, and regulation [67].

The main application of this technology is for prophylactic vaccines; however, their valuable properties allow them to be used as nanocarriers in drug and gene therapy, as scaffolds for bioimaging, and for the synthesis of bionanomaterials [72,73,74]. Regarding its uses in plants and plant cells, in addition to being vehicles for the transport of RNAi, it is used to carry the CRISPR/Cas complex as a DNA-free gene editing system [75].

### 4.3. Polyplex Nanoparticles

The term polyplex nanoparticles refers to the encapsulation of genetic material in polymeric particles. The polymers used are polycations that electrostatically interact with negatively charged nucleic acid molecules. Thus, neutralization of charges (phosphodiester groups) and the consequent compaction in a colloidal complex occur. It is also stabilized by hydrogen bonding interactions between the components [76]. The final properties of the polyplex depend on the physicochemical characteristics of the pristine polymers and the resulting nanoparticle features: size, surface charge, polydispersion, and hydrophilicity [77].

There is a large number of polymers used for the manufacturing of polyplexes, and a limited list of the most commonly used polymers includes poly(ethyleneimine) (PEI) [77], poly-L-lysine (PLL), poly(amidoesters) (PAE) [78], polyamidoamine (PAMAM), poly(2-dimethylaminoethyl methacrylate) (PDMAEMA) [79], polyethylene glycol (PEG), and chitosan [80], as well as copolymeric combinations such as phosphorylcholine-modified polyethyleneimine (PEI) PEGylated PEI-based, PEGylated poly(dimethylaminomethyl methacrylate) containing folate, PEI-graftedα, β-poly(N-3-hydroxypropyl)-DL-aspartamide, galactose-modified trimethyl chitosan-cysteine-based, and others [81]. Theoretically, any positively charged polymer could form a polyplex, of particular interest being biodegradable polymers such as PEG, poly(glutamic acid) (PGA), poly(caprolactone) (PCL), poly(D,L-lactide-coglycolide) (PLGA), poly(lactic acid) (PLA), N-(2-hydroxypropyl)-methacrylate copolymers (HPMA), polystyrene-maleic anhydride copolymer, and poly(amino acids) (PAAs) [81]. Polycations can also be synthesized by ring-opening polymerization controlling the resulting charge [82].

The synthesis methods of polyplexes are very diverse, defined by the raw material and the application. There are promising methodologies, such as the three-dimensional hydrodynamic focusing (3D-HF) technique supported by microfluidic devices. It seeks to optimize the properties and the resulting system’s effectiveness; since conventional methods can become a limitation since some of them involve an organic-aqueous interface, shear stress, or pH and temperature levels that can damage the genetic material [78]. Another limitation is the reduced biocompatibility and biodegradability of some polymers, as well as their cytotoxicity, requiring the production of derivatives to cope with these disadvantages [83].

Nevertheless, they are a widely used transport mechanism for genetic material because they manage to prevent the degradation of genetic material caused by extracellular enzymes and precisely direct the load to the target cells, increasing the effectiveness of gene expression systems [78]. Nanoparticle uptake occurs by endocytosis involving interactions with cell membrane glycoproteins and the formation of the endosome, followed by its release into the cell cytoplasm and further translocation to the target organelles [76].

Its main application is as a nonviral vector for DNA transfer in gene therapy, including regenerative medicine [78], AIDS, cancer, and hereditary disorder treatments [79]. It has also been used for transfection and editing of genomes in plants [84,85], as well as for the gene silencing systems mentioned above aimed at disease and pest control [22,47,52,86,87].

### 4.4. Bioclays

Bioclay systems are based on 10–100 nm nanoclay particles able to transport selected compounds such as nucleic acids. Nanoclays are stratified aluminosilicates of a single (0.7 nm thick) or double layer (1 nm thick) with variable plasticity and swelling capacity. Its chemical formula is (Ca, Na, H) (Al, Mg, Fe, Zn)_2_(Si, Al)_4_O_10_(OH)_2_-xH_2_O. There are both natural and synthetic materials, and their structures consist of alternating tetrahedral SiO_2_ and octahedral AlO_6_ sheets with varying ratios [88,89].

Its properties depend on the nature of the atoms on the surface and the exchangeable cations between layers. Clays are negatively charged due to the substitution of Al^3+^ or Mg^2+^ ions by Si. Consequently, the surface of the nanolayer is hydrophobic due to Si-O covalent bonds; however, the addition of exchangeable hydrophilic cations can change the charge to positive [88].

The most commonly used raw materials are bentonite, hectorite, montmorillonite, kaolinite, laponite, halloysite, laponite, sepiolite, saponite, and vermiculite. They are low-cost, nontoxic, and charge versatile vehicles [89,90].

Surface functionalization is frequently performed to adapt them to specific applications. Processes such as silylation prevent the aggregation of clays through covalent modification of the surface. For example, modified montmorillonite shows improvement in the polymer mechanical performance as well as in water absorption, cationic exchange, and ionic retention capacities. Other clays, such as sepiolite, are functionalized by grafting organosilane due to its many silanol groups on the surface, making them excellent heavy metal removers. Halloysite is modified mainly by covalent grafting of organosilane via condensation with hydroxyl groups [91]. Synthetic clays, such as Laponite^®^ (phyllosilicate composed of layered synthetic silicate), are obtained from inorganic mineral salts and are superficially modified by ion exchange or covalent linkage [91].

Their applications mainly cover materials science, chemistry, physics, and biology, where they become strategic materials for developing smart nanoarchitectures. Among the most documented uses are drug delivery, environmental remediation, wastewater treatment, and food packaging [91,92,93]. Plant biotechnology has also been used for the manufacture of nanocomposites to improve resistance properties in wood [94,95], vehicles for the controlled dosage of nutrients, and regulating soil properties [96,97,98]. It is relevant to mention the transformation of desert soils into cultivable fields through liquid nanoclay [99].

## 5. Regulatory Approaches

The time and cost associated with obtaining the data for a registry of a biomolecule such as dsRNA can be low compared with that of a conventional pesticide of 4 versus 12 years and USD 3–7 million versus USD 280 [10]. An important consideration is that dsRNA generally has low environmental persistence in soil, sediment, and water [100,101]. The biomolecule shows a record of safe consumption of short and long RNAs in the diet from food and lacks oral immunostimulation [11]. Another positive input when regulating this technology is the technical discussion that has occurred over the last decade, where the USEPA and OECD (Organization for Economic Co-operation and Development) proposed using and adapting the existing plant protection product norms and procedures as described next. The United States Environmental Protection Agency (EPA) has analyzed addressing this technology based on problem formulation for human health and ecological risk assessment. At the same time, OECD proposed using risk assessment to evaluate the toxicity profile and exposure of the molecule by adapting the current regulatory framework for small-molecule agrochemicals as a general framework for dsRNA-based agricultural products and proposed taking into consideration the experience gained through the review of dsRNA-based GE crops. The EFSA literature review on GM plants is a document to be taken into consideration as well. A summary is presented next in Table 3.

## 6. Conclusions and Future Perspectives

RNAi technology is a powerful and versatile alternative for pest and disease control in crops. Its use in the agricultural field extends to viruses, bacteria, fungi, insects, nematodes, and plants. It grows steadily with other complementary technologies, such as the recombinant production of RNAi in vectors, transgenesis, and micro/nanoencapsulation of candidate si/dsRNA. The main issue preventing its adoption in the past was the cost of production and stability. The cost of production is getting lower with the development of new technologies, while stability encapsulation strategies provide a solution to avoid degradation.

Encapsulation of RNAi in liposomes, virus-like particles, polyplex nanoparticles, and bioclay has gained relevance in the last decade because they confer protection against degradation. Improving its stability has been a challenge during the development of this technology. Naked dsRNAs is easily degraded because of environmental exposure or the action of enzymes and the pH level of the target organism. Encapsulation provides stability to dsRNA and sometimes also improves cell uptake. Some of the materials used for encapsulation provide additive effects for pest control; however, most of them are innocuous, biodegradable, and stable in multiple chemical environments, favoring the controlled release of RNAi. Our review found multiple reports of this technology applied mainly for the control of insects, where the predominant administration mechanism is the oral route using SIGS or the application of encapsulation on baits.

Candidate genes for targeted silencing coincide in essential genes related to enzymes involved in cell division (e.g., *CDC27*, *gTub23C*, *TVXl*, *Cep89*), cell transport (e.g., *KIF*, *alpha COP*), structure formation (e.g., *ChSB*, *act-2*, *MEGF10*, *A5C*), ionic balance and nutrient absorption (e.g., V-ATPase, vha26), producing mortality in the target species.

Current regulatory approaches to products developed with RNAi technology focus on assessing their risk from different approaches. However, based on the characteristics of these biomolecules and their proven safety in nontarget organisms, a favorable position is predicted for the use of this technology in agriculture, where the will to regulate is optimistic regarding their economic and environmental advantages and low risks associated with human health. The regulatory landscape can allow the safe adoption of this technology with current decision-making based on risk assessment. However, a harmonized approach will soon be needed to enable adoption and avoid trade disruptions.

Together with the positive approaches to regulation, the emergence of more interdisciplinary alternatives that combine gene silencing by RNAi is also expected. For instance, the induction of resistance in crops by elicitation and metabolic control methods, using the strengths of both. Following this approach, we are developing a technology that uses elicitor nanoparticles made of natural polymers to induce defense in plants, which will also carry a double control mechanism based on RNAi to unblock inhibitors of systemic defense against systemic defense pathogenic species of the genus *Fusarium*. There is also interest in the scientific community to produce multiple knockdowns that protect systemically against a consortium of pathogens under the same application. Another challenge for this technology is to keep reducing production costs, for which biotechnology is emerging as one of the main allies to improve profitably on a large scale.

## Figures and Tables

**Table 1:** Potential targets for spray-induced gene silencing (SIGS) in plants.

Target	Experimental Evidence	Target Genes	Reference
Virus	dsRNA + clay resulted in BCMV virus resistance for 20 d	*Nib* and *CP* genes of *BCMV*	[24]
	TMV Tobacco virus resistance for 7–20 d	*CP*, *P126*, *RP* of *TMV*	[25,26]
Fungi	Inhibits *Botrytis cinerea* disease	*DCL1*, *DCL2* of *Botrytis cinerea*	[27]
	Efficiently inhibited *Fusarium graminearum*	*CYP51A*, *CYP51B*, *CYP51C* of *F. graminearum*	[28]
	*Sclerotinia sclerotiorum/Botrytis cinerea*	mRNA splicing, ribosome biogenesis, *protein disulfide oxidoreductase*, peroxisomal protein	[27]
	*Fusarium asiaticum*, *Botrytis cinerea*, *Magnaporthe oryzae*, *Colletotrichum truncatum*	*β_2_-tubulin*	[29]
	*Fusarium oxysporum f. *sp.* cubense *and* Mycosphaerella fijiensi*, *Fusarium*	*adenylate cyclase*, *DNA polymerase alpha subunit*/*delta subunit*/*CYP51*	[30,31]
Nematodes	*Caenorhabditis elegans*, *Radopholus similis*, *Meloidogyne artiellia*, *Meloidogyne incognita*, *Globodera pallida*	Several genes such as *16D10 peptide*, *chitin synthase*, *xylanase*, *glutathione-S- transferase*, *FMRF-like peptides*	[18,32,33,34,35,36]
Insects	Coleopterans are highly sensitive, Hemiptera, Orthoptera, Diptera, Hymenoptera, and Lepidoptera have different responses.	Several genes such as salivary protein *C002*, *16D10*, *αCOP*, *Cytochrome P450*, *Acetylcholinesterase*, *ABC transporter*, *β-actin*, *chitin synthase B*	[37,38,39,40,41,42]
Endogenous plant genes	Arabidopsis, Tobacco, poplar, rice	Transgenes/*CHS*/*EPSPS*/*STM*/*WER*/*MYB1*/*WRKY23*	[43]

BCMV: potyvirus bean common mosaic virus, TMV: tobacco mosaic virus.

**Table 2:** Summary of crop protection applications using an encapsulation system for RNAi delivery.

Encapsulation System	Potential Crop Protection Application	Strategy	Reference
Guanylated 2-(aminoethyl) methacrylate (AEMA)/dsRNA polyplex nanoparticles.	Insecticide induces decreased feeding in Lepidopteran larvae (*Spodoptera exigua*), then promoting weight loss, developmental halt, and mortality.	Increases RNAi efficiency in targeting the essential gene *chitin synthase B* (*ChSB*), while preventing the degradation of dsRNA in the alkaline gut of insects and enhancing its cellular uptake in midgut cells.	[47]
poly-[N-(3-guanidinopropyl) methacrylamide] (pGPMA)/dsRNA interpolyelectrolyte nanocomplex.	Ingestion insecticide regulates gene silencing in Lepidopteran larvae (*Spodoptera frugiperda*), increasing mortality from starvation and growth stunting.	Increased internalization and protection of dsRNA in insect cells, decreasing the accumulation of target mRNA due to the knockdown of genes related to vital functions such as nutrient absorption (*sfVATPase*), intracellular transport (*sfKIF*), and cell division (*sfCDC27*).	[52]
Chitosan/dsRNA polyplex nanoparticles	Nematicide can homogeneously enter the nematode’s body (*Caenorhabditis elegans*) through noncanonical endocytotic pathways and attack specific genes. The combined effect decreases the development of the nematode by the action of the chitosan vehicle.	Increases RNAi efficiency of gene knockdown throughout the whole body of the nematode by introducing intact dsRNA through the Clathrin-mediated endocytosis pathway, which is different from the canonical pathway (sid-1 and sid-2) in the study model. Furthermore, chitosan was shown to effectively decrease myosin gene expression, which is critical for the growth and reproduction of the model nematode.	[22]
Chitosan/dsRNA polyplex nanoparticles	Insecticide against Lepidopteran larvae (*Spodoptera frugiperda*) acts on genes related to the apoptosis pathway, inducing growth impairment and larval mortality.	Improves RNAi efficiency through the protection of dsRNA from degradation by intracellular and intercellular RNases. It also reduces the accumulation of dsRNA in the endosome while favoring its transport to the cytoplasm, where the formation of siRNAs is promoted, producing knockdown of apoptosis-related genes (*iap*).	[45]
Layered double hydroxide (LDH) clay nanosheets/dsRNA	Develop a topical product that induces viral resistance in plants (against PMMoV and CMV) using dsRNA absorption technology in clay nanosheets (Bio-Clay).	Increased persistence of the topical treatment due to strong adhesion of dsRNA in the vehicle (LDH) and with the leaves. It also allows the controlled release of the biomolecule and confers protection against environmental degradation while favoring the internalization of dsRNA in the plant.	[44]
Lipofectamine 2000 liposomes/dsRNA.	Insecticide against Diptera of the genus Drosophila (*D. melanogaster*, *D. sechellia*, *D. yakuba*, and *D. pseudoobscura*) acting by ingestion. It attacks essential genes of development through knockdown management.	Promotion of dsRNA internalization in insects through encapsulation protection, increasing silencing efficiency by promoting more significant RNAi accumulation in larvae. Knockdown of the genes of the *VATPase* (gut lumen pH stabilizer associated with nutrient uptake) and *gTub23C* (mitosis-related g-tubulin protein, essential for microtubule organization).	[55]
Lipofectamine 2000 liposomes/dsRNA.	Specific insecticide against larvae and adults of *Drosophila suzukii* combining synergic effect of multiple gene knockdown. Oral administration route.	It facilitates uptake in the insect’s gut. It causes significant mortality in larvae and adults by the reduction in transcript levels of essential genes *rps13* (housekeeping), *alpha COP* (coatomer subunit for trans-organelles transport), and *vha26* (subunit of the vacuolar ATPase). The synergistic action of knockdown of the *rps13* and *alpha COP* genes significantly increases mortality in the insect.	[53]
Liposomes/dsRNA	Oral insecticide for the control of nymphs of *Euschistus heros* (hemiptera: pentatomidae), which is one of the main soybean pests in the field.	Protection of dsRNA against degradation promoted by the ribonuclease action of insect saliva. Enhanced silencing activity of target genes *vATPase*A (V-type proton ATPase catalytic subunit A) and *act-2* (muscle actin).	[56]
Recombinant Flock House Virus FHV/dsRNA	Recombinant insecticide based on a viral vehicle transporting dsRNA silencers of essential genes in *Drosophila melanogaster*. For potential massive application in other species susceptible to FHV infection.	Use of the insect cell machinery to assemble infective recombinant FHV virions that carry target sequences for the production of dsRNA when replicating in cells. Thus, virions protect the sequences responsible for silencing the *rps13* (housekeeping), *alpha COP* (coatomer subunit for trans-organelle transport), and *vha26* (subunit of the vacuolar ATPase) genes while at the same time favoring dispersal in insects. It simulates natural viral infection.	[54]
Virus-Like Particles (VLP)/dsRNA	Oral insecticide for the control of ants of several genera (*Solenopsis invicta* (fire ants), *Camponotus pennsylvanicus* and *Camponotus floridanus* (carpenter ants), *Linepithema humile* (Argentine ants), *Tapinoma sessile* (odorous ants), *Tetramorium caespitum* (pavementom ants), and Monstrous ants) pharaonis (pharaoh ants); inducing the silencing of physiological genes required for the survival of the colony.	Recombinant production in *E. coli*, which through specific plasmids manufactures capsid proteins of bacteriophages Qß and MS2 and inducible RNAi precursor sequences. The packaging of dsRNAs in VLPs protects them from degradation by nonspecific environmental organisms and the intestinal RNases of the target organism. It also favors its absorption by lining the gut cells. The silenced genes are related to the viability of the colony, for example, the induction of sterility and individual mortality. VLP carrying dsRNA is sprayed on the ground for spot application or incorporated into the bait. Target genes include *VgR* (vitellogenin receptor protein), *TVXl* (telomerase variant XI protein), *PBAN* (pheromone biosynthesis activating neuropeptide), *PBANR* (pheromone biosynthetic activating neuropeptide receptor), *WLS* (wntless protein), *MEGF10* (multiple epidermal growth factor-like domain proteins 10), *CHCP* (clatherin heavy chain protein), *CDC7* (cell division cycle 7-related protein), *Cep89* (centrosomal protein 89 kdal), *PSMBl* (beta subunit of the type-1 proteosome), *A5C* (actin 5C protein), *ATPSD* (ATP synthase delta subunit); as well others related with anamorsin, beta actin, and Csp9 proteins	[46] WO2017/136353Al for APSE RNA Containers (ARCs)
Ribonucleoprotein particle (RNP)/dsRNA	Insecticide for control of cotton boll weevil (*Anthonomus grandis*) adults.	Developing a protection and stability system for dsRNA avoids degradation by nucleases in the insect’s gut and favors rapid cellular incorporation. The above is based on a chimeric protein PTD-DRBD (peptide transduction domain–dsRNA binding domain) combined with dsRNA. This type of resulting protein is known as cell-penetrating peptide (CPP).	[51]

**Table 3:** Regulatory approaches.

Regulatory Agency	Proposal	Reference
EPA	Propose using Problem Formulation- Risk assessment	[102]
European Food Safety Authority (EFSA)	Do not directly address the spray products, but a literature review focused on RNAi-based GM plants and Risk Assessment	[86]
OECD	Proposed using risk assessment to evaluate the toxicity profile and exposure by using the current regulatory framework for small-molecule agrochemicals as a general framework for dsRNA-based agricultural products. Proposed using the experience with the review of dsRNA-based GE crops	[11]

## Data Availability

Not applicable.
